# 1542. Impact of Subcutaneous Administration Sites on the Clinical Pharmacokinetics of Lenacapavir, a Long-Acting HIV Capsid Inhibitor: Does Body Site Matter?

**DOI:** 10.1093/ofid/ofad500.1377

**Published:** 2023-11-27

**Authors:** Allen K Lat, Aryun Kim, Haeyoung Zhang, Stephen Lin, Eva Mortensen, Ramesh Palaparthy, Renu Singh

**Affiliations:** Gilead Sciences Inc, Burlingame, California; Gilead Sciences, Inc., Foster City, California; Gilead Sciences Inc, Burlingame, California; Gilead Sciences Inc, Burlingame, California; Gilead Sciences Inc, Burlingame, California; Gilead Sciences Inc, Burlingame, California; Gilead Sciences Inc, Burlingame, California

## Abstract

**Background:**

Lenacapavir (LEN) is currently approved for multidrug-resistant HIV-1 infection in combination with other antiretrovirals for heavily treatment-experienced individuals. Mean trough concentrations >15.5 ng/mL, which is the inhibitory quotient-4 (IQ4; 4-fold in-vitro protein binding-adjusted 95% effective concentration) are associated with high rates of HIV-1 suppression. LEN dosing regimens consist of a subcutaneous (SC) 927 mg injection (2 x 1.5 mL) every 6 months along with oral loading. With SC injections, it is common practice to rotate injection sites (within the same or at a different body site) to mitigate injection site reactions while also providing flexibility in dosing. However, SC LEN is approved to be administered in the abdomen only. This study investigated the impact of alternate injection sites on the pharmacokinetics (PK) and safety/tolerability of SC LEN.

**Methods:**

This was a Phase 1, open-label, parallel design, single dose, multicohort study in healthy participants. Participants were enrolled into a thigh (TH), upper arm (UA), or abdomen (AB) cohort (N=10 per cohort) and received a single approved SC dose of 927 mg. Plasma PK samples were collected on Day 1 over 0–216 hours post dose, followed by a weekly or biweekly sample over Days 15–210, then monthly until Day 270 and were analyzed for LEN using validated bioanalytical methods. PK of LEN was characterized by noncompartmental analysis, and LEN plasma exposures in the TH and UA cohorts were descriptively compared to AB as the reference.

**Results:**

Mean [CV%] C_max_ and AUC_tau_ (tau = 6 months) were similar between TH vs AB (63.4 [53%] vs 60.6 [38%] ng/mL; 6020 [53%] vs 6360 [35%] days*ng/mL respectively). For UA, mean C_max_ (86.0 [56%] ng/mL) and AUC_tau_ (7760 [42%] days*ng/mL) were higher than AB by 42% and 22% respectively, which were not considered clinically significant. Mean C_tau_ [CV%] for TH, UA, and AB were higher than IQ4 (26.8 [62%], 21.3 [50%], and 31.9 [42%] ng/mL, respectively). SC LEN was well-tolerated in TH, UA, and AB with no serious adverse events.

**Conclusion:**

TH and UA cohorts achieved similar or higher overall exposures compared to the reference AB, with mean C_tau_ exceeding the efficacy target of IQ4. This study supports TH and UA as potential alternate administration sites for SC LEN in future studies.

**Disclosures:**

**Allen K. Lat, PharmD**, Gilead Sciences, Inc: Grant/Research Support **Aryun Kim, PharmD**, Gilead Sciences, Inc: Company employee|Gilead Sciences, Inc: Stocks/Bonds **Haeyoung Zhang, PharmD, PhD**, Gilead Sciences, Inc: Employee|Gilead Sciences, Inc: Stocks/Bonds **Stephen Lin, BA**, Aurinia Pharmaceuticals Inc: Stocks/Bonds|Biodexa Pharmaceuticals: Stocks/Bonds|Clearside Biomedical: Stocks/Bonds|Gilead Sciences, Inc: Company employee|Gilead Sciences, Inc: Stocks/Bonds **Eva Mortensen, MD, MS**, Ascendis: Stocks/Bonds|Johnson and Johnson: Stocks/Bonds|Merck: Stocks/Bonds|Moderna: Stocks/Bonds|Novartis: Stocks/Bonds|Pfizer: Stocks/Bonds|Teva: Stocks/Bonds **Ramesh Palaparthy, PhD**, Gilead Sciences, Inc: Employee|Gilead Sciences, Inc: Stocks/Bonds **Renu Singh, PhD**, Gilead Sciences, Inc: Employee|Gilead Sciences, Inc: Stocks/Bonds|Pfizer: Stocks/Bonds

